# Association between Eating Out and Socio-Demographic Factors of University Students in Chongqing, China

**DOI:** 10.3390/ijerph14111322

**Published:** 2017-10-30

**Authors:** Ping Hu, Tingting Wu, Fan Zhang, Yan Zhang, Lu Lu, Huan Zeng, Zu-min Shi, Manoj Sharma, Lei Xun, Yong Zhao

**Affiliations:** 1 School of Public Health and Management, Chongqing Medical University, Chongqing 400016, China; m18323165365@163.com (P.H.); m17749945967@163.com (T.W.); ava11@126.com (F.Z.); zyan325@126.com (Y.Z.); kkllu001@126.com (L.L.); zenghuan586@aliyun.com (H.Z.); leixun521@163.com (L.X.); 2 Research Center for Medicine and Social Development, Chongqing Medical University, Chongqing 400016, China; 3 The Innovation Center for Social Risk Governance in Health, Chongqing Medical University, Chongqing 400016, China; 4 Chengdu Blood Center, Chengdu 610041, China; 5 Adelaide Medical School, University of Adelaide, Adelaide 5005, Australia; zumin.shi@adelaide.edu.au; 6 Behavioral & Environmental Health, Jackson State University, Jackson, MS 39213, USA; manoj.sharma@jsums.edu

**Keywords:** eating out, frequency, socio-demographic, university students

## Abstract

(1) *Objective*: We aimed to explore the current situation of eating out and the association with socio-demographic factors of university students in Chongqing, China. (2) *Methods*: We used self-administered questionnaires to collect information. There are 14 universities in Chongqing; four (Chongqing Medical University, Chongqing University, Chongqing Normal University, and Chongqing University of Science & Technology) were randomly selected. In each selected university, two disciplines were randomly selected. (3) *Results*: 4595 university students participated in the study. The frequency of eating out was relatively high. The frequency of eating out among females was higher than that among males during weekdays. The two main reasons for eating out were having an opportunity to meet friends (56.0%) and improving diet (39.6%). Bistros (61.7%) and hot-pot restaurants (41.1%) were the favorite places for eating out. Only 36.0% of the participants said they considered nutrition and food safety when selecting restaurants. The majority of the participants demonstrated a high demand for nutrition and food safety knowledge when eating out (77.7%). (4) *Conclusions*: The higher the monthly living expenses were, the higher the frequency of eating out was. An intervention strategy to reduce the frequency or change the behavior of eating out should be formulated by considering the students’ perspectives.

## 1. Introduction

A steady increase in the frequency of eating out of the home has been observed in many countries over the last few decades [1,2,3,4,5], and this trend is likely to continue. Eating out of the home is becoming particularly popular among young adults. A study in the United States showed that young adults consume approximately 40% of their total daily energy away from home [6]. In China, about 15% of the residents eat outside of their home every day [7].

Eating out of the home is often associated with increased intake of energy [8,9,10], fat, and sugars [11,12], and this increased intake results in the reduced consumption of dietary fiber and vegetables, low nutrient density [13], and poor diet quality [9,14,15]. Eating out is one of the main obesity-promoting behaviors [16].

Obesity is a global public health concern. One in three adolescents is overweight, and this scenario dramatically increases in low-income and middle-income countries [17]. Overweight and obesity are associated with an increased risk of several chronic diseases, including type 2 diabetes, hypertension, and cardiovascular disease [18,19].

Adolescent nutrition is a predictor of adult nutrition status [20] and provides an important window of opportunity to prevent diet-related non-communicable diseases, which can be acquired later in adulthood [21]. After entering the university, young adults become increasingly independent and develop their identities in a different social environment; this situation often leads to different food choices and poor dietary habits [22].

Few studies have focused on the current situation of eating out and the correlation of socio-demographic profiles to eating out frequency in Chongqing, China. As such, the objectives of our study are to explore the current situation of eating out and its association with the socio-demographic factors of university students in Chongqing, China.

## 2. Materials and Methods

### 2.1. Study Design and Participants

In April 2014, a cross-sectional study was conducted in a college town in Chongqing, China. Among 14 universities, four (Chongqing Medical University, Chongqing University, Chongqing Normal University, and Chongqing University of Science & Technology) were randomly selected. In each selected university, two disciplines were randomly selected. Then, the junior classes (first and second year students) in the two departments in each university were randomly selected. All the students in the selected classes were invited to participate in the study. A total of 4595 students participated in our study.

At present, most universities in China still carry out the traditional two-semester system: each academic year is divided into fall (September to January) and spring (March to July) semesters [23]. Therefore, April is a stable school time—not the beginning of the term and not the end of the term. In this period, there is a high risk of certain kinds of infectious diseases, and the food safety risks of eating out are higher [24,25], so it was decided that eating out behavior during this period was most worth studying.

### 2.2. Investigation Method and Outcome Measurements

#### 2.2.1. Investigation Method

With each class representing one unit, students were contacted in their classrooms before or after lectures. After obtaining informed consent, a self-reported questionnaire was provided to the participants for data collection. This process took approximately 10–15 min. Afterwards, a quick review of the questionnaires was performed by the investigators to check for completion.

#### 2.2.2. Outcome Measurements

The questionnaire was self-administered and specifically designed for the target population. The final version was completed after a pilot investigation (127 students participated in the pretest) and repeated discussions with experts. The instrument had acceptable face and content validity and contained both open- and close-ended questions that were grouped into three sections.

The three sections included informed consent; basic information and eating out behavior. The informed consent is a letter for participants to show the purpose of this study and to let them participate the study voluntarily. The basic information included the participant’s age, gender, height, weight, residence(urban/rural), father’s education, mother’s education, father’s occupation, mother’s occupation, monthly living expenses, and monthly accommodation fee, as well as where the participant is living while they study and where they go during holidays. And the eating out behavior included 11 questions regarding (1) frequency of eating out, (2) cost of eating out per person per meal, (3) reason for eating out, (4) which types of restaurants chosen, (5) what is considered when choosing restaurant, (6) how information on restaurants is most often obtained, (7) health effects of eating out for a long time, (8) potential risks of eating out for a long time, (9) the reason for these potential risks, (10) whether knowledge on eating out is sought and, if so, how, and (11) whether an increase in knowledge on eating out is desired and, if so, how. The options for these questions in the third section were as follows: (1) 0 time; 1 time; 2 times; 3 times; 4 times; 5 times or more, (2) <10; 10–20; 20–50; 50–100; >100, (3) improved diet; miss canteen meal time; family dinner; friend dinner; outside when it is time to eat; other, (4) bistro (small restaurant); street food; Western-style fast-food restaurant; Chinese fast-food restaurant; hot-pot restaurant; upscale Chinese food restaurant (average cost of >70 Yuan); supermarket or convenience store; other, (5) price; nutrition and food safety; characteristic of dishes; service; environment; location; characteristic; other, (6) recommendations from others; leaflet; new media; group-buying; food magazine; TV show; chance encounter; other, (7) good; bad; nothing; not always, (8) too much unhygienic food; high energy; too much meat; too much spicy food; too much fried and barbecue food; high salt; too much sweet too much carbonated beverage; drinking too much; other, (9) unhygienic food; lack of nutrition and food safety knowledge; lack of knowledge of eating out; the limitations of food type; preference; family eating habits; picky eating; economic factors; restriction of eating; other, (10) yes (publication; TV; professional lecture and class; guidance of family or friend; internet; other); no; cannot remember, (11) yes (internet; professional lecture and class; guidance of family; TV; publication; other); no; do not know.

The frequency of eating out per week for the previous month was considered in terms of weekdays and the weekend. It was assessed by the question, “how often did you eat out per week in the last month?”

The participants were asked to report their height and weight. Body mass index (BMI) was calculated as the ratio of weight (kg) to the square of height (m). Participants with BMI ≥ 24 kg/m^2^ and <28 kg/m^2^ were classified as overweight, and those with BMI ≥ 28 kg/m^2^ were classified as obese according to Chinese criteria [26].

### 2.3. Quality Assurance

All investigators for the study were recruited via interview to join the investigation team at the start of each term. The major teachers of these students gave them general training once or twice a month and specialized training prior to the implementation of each survey. Only investigators familiar with the approach, objectives, and methodology of the research as well as those who had experience in handling potentially sensitive issues were allowed to conduct the survey.

### 2.4. Ethics Statement

This project was reviewed and approved by the Ethical Committee of the Chongqing Medical University (record number: 2013036) and was registered in the Chinese Clinical Trial Registry (Number: ChiCTR-OCH-14004861). Written informed consent was obtained from all participants. The participants were informed that they could withdraw from the study at any stage.

### 2.5. Statistical Analyses

The data in the questionnaires were checked carefully before being encoded in the database with the Epidata 3.1 software (Myatt, Denmark). All entries were double-checked to avoid errors. Strict sorting was conducted, followed by data cleaning. Statistical analyses were performed with a statistical analysis system software (version 9.0; SAS Institute, Cary, NC, USA). Descriptive data were expressed as mean ± standard deviation (SD) or proportions. The chi-square test was utilized to test the differences of the categorical variables. Logistic regression analysis was implemented to analyze the factors associated with the frequency of eating out. All statistics were analyzed through a two-sided test; a *p*-value that is less than or equal to 0.05 was considered statistically significant.

## 3. Results

### 3.1. Demographic Characteristics of the Study Sample

Among the 4595 junior university students approached, 359 did not agree to complete the survey or did not fill in the questionnaires completely; they were thus deemed unqualified. The final sample consisted of 4236 (92.2%) students.

Table 1 shows the demographic characteristics of the study population. A total of 1749 females and 2487 males were included in the study. The mean age of the participants is 19.8 (SD 1.2) years. A total of 214 males (12.2%) are overweight or obese, 54.9% of the participants are from rural areas, and 40.6% and 39.4% have fathers and mothers who finished middle school, respectively. The monthly living expenses of 51.1% of the participants range from 800 to 1200 Yuan, and the monthly accommodation fee of 63.7% of the participants is below 600 Yuan. The majority (93.2%) of the participants live in their school during the study, and this figure decreases to 63.7% during holidays.

### 3.2. Current Situation of Eating Out among University Students

#### 3.2.1. Frequency of Eating Out

As shown in Table 2, 26.3% of all the participants had eaten out weekly in the last month and were more than three times likely to do so on weekdays. The frequency among females and males exhibited no difference. A total of 27.4% of the participants reporting eating out on weekends more than three times, and the frequency among females was higher than males (*p* = 0.04). Only 12.4% of the participants reported no eating out on weekends in the last month (data not shown in Table 2).

#### 3.2.2. Reasons for Eating Out

The top three reasons of the participants for eating out were having an opportunity to meet a friend (total: 56.0%; male: 56.8%; female: 55.6%), improving diet (total: 39.6%; male: 36.5%; female: 41.7%), and being outside when it is time to eat (total: 28.6%; male: 20.0%; female: 34.7%). The order did not change after being grouped according to gender.

#### 3.2.3. Types of Restaurant

All participants were asked which type of restaurants they often select. Bistros (small licensed restaurants) were the favorite eating out place of females (64.5%). Hot-pot restaurants (41.5%) and street food vendors (33.2%) were also preferred. Similar to the females, the males also preferred bistros (61.7%), hot-pot restaurants (41.1%), and street food vendors (28.7%).

#### 3.2.4. Concerns in Selecting Restaurants

**Figure 1:**
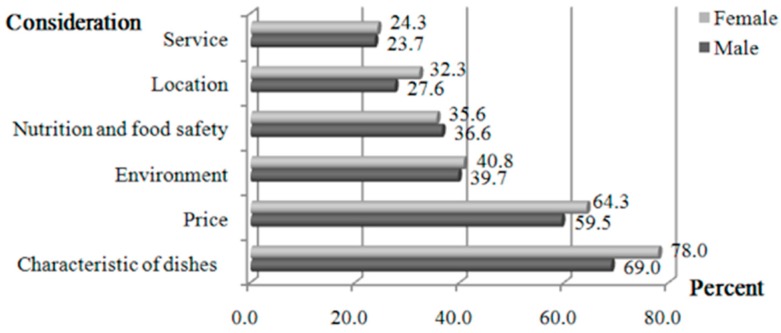
What are you thinking about when you can choose restaurant?

In regard to the question “what do you consider when you select restaurants”, both males and females (69.0% and 78.0%) paid particular attention to the characteristic of the dishes, 59.5% and 64.3% of males and females considered the price, and 39.7% and 40.8% of males and females focused on the environment. Only 36.0% (male: 36.6%; females: 35.6%) paid attention to nutrition and food safety (Figure 1).

#### 3.2.5. Potential Risks of Eating Out for a Long Time

**Figure 2:**
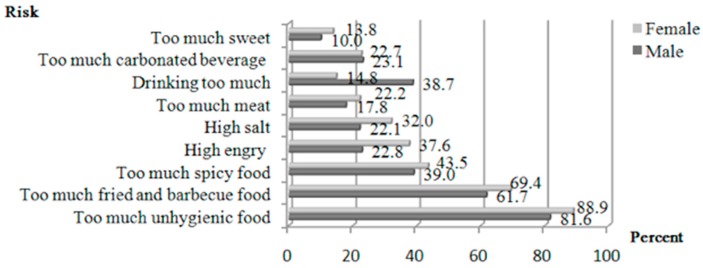
The potential risks of eating out for a long time.

Two-fifths (total: 41.7%; males: 36.8%; females: 45.1%) of the participants said that frequently eating out is harmful to their health. For both males and females, the top three potential risks were eating too much unhygienic food (total: 85.9%; males: 81.6%; females: 88.9%), eating too much fried and barbecued food (total: 66.2%; male: 61.7%; female: 69.4%), and eating too much spicy food (total: 41.7%; males: 39.0%; females: 43.65%). For males, the additional risks were drinking too much alcohol (38.7%) and carbonated beverages (23.1%). For females, the additional risks were high energy (37.6%) and high salt (32.0%) intakes (Figure 2).

#### 3.2.6. Possible Cause of Potential Risks of Eating Out for a Long Time

With respect to the possible reasons for these perceived risks, the majority of the participants (total: 86.1%; males: 82.6%; females: 88.7%), especially females, selected “unhygienic food”. Of the participants, 52.2% selected “lack of nutrition and food safety knowledge on eating out” (females: 54.7% and males: 48.6%). One-third of the participants (total: 33.4%; males: 30.1%; female: 35.7%) selected “picky eating”. One-fourth of the participants (total: 24.8%; males: 22.8%; females: 26.2%) selected “economic factors”.

#### 3.2.7. Requirement of Nutrition and Food Safety Knowledge on Eating Out

The results of the investigation showed that the majority of the participants had a high demand for nutrition and food safety knowledge on eating out (total: 77.7%; males: 75.6%; females: 79.2%). With regard to the means of obtaining knowledge, two-thirds (total: 66.1%; males: 66.7%; females: 65.7%) of the participants selected “Internet”, half of the participants (total: 48.8%; male: 44.2%; female: 51.9%) selected “publication”, and one-third selected “professional lecture and class” (total: 40.0%; males: 42.0%; females: 39.3%). The rest cited “TV” (total: 31.4%) and “guidance of family or friend” (total: 22.9%).

### 3.3. Factors That Affect the Eating Out Frequency of Chinese University Students

Frequent eating out was considered in terms of weekdays and the weekend (both >3 times/week). Several factors were considered in modeling the determinants of frequent of eating out (age, BMI, residence, parent’s education, monthly living expenses, monthly accommodation fee, living place, and so on). Monthly living expenses, monthly accommodation fee, and living place during holidays affected the frequency of eating out on both weekdays and the weekend, as observed in the logistic regression analysis (Table 3). During weekdays, the higher the monthly living expenses were, the higher the frequency of eating out was (*p* < 0.001). The higher the monthly accommodation fee was, the higher the frequency of eating out was (*p* < 0.001). Those who rented houses were more likely to eat out than those who lived in their own homes (OR = 1.654, *p* < 0.001). No significant difference was observed between those who lived in a dormitory and those who lived in their own home (*p* = 0.149). During weekends, the factors of monthly living expenses and the monthly accommodation fee were consistent. Those who lived in rented houses were more likely to eat out (OR = 2.362, *p* < 0.001), and those who lived in their own houses were more likely to eat out than those who lived in dormitories (OR = 0.823, *p* = 0.029).

## 4. Discussion

The frequency of eating out was high during weekdays and the weekend. The prevalence of eating out in this study is higher than that in the results of the 2002 China National Nutrition and Health Survey [26] and the 2011 China Health and Nutrition Study [27]. The frequency of eating out among females was higher than that among males during weekdays; no significant difference was observed between females and males during weekends. Previous studies have revealed that food not prepared at home constitutes a higher amount of men’s diets compared with women’s diets [8,28,29,30]; however, one study reported that no significant difference was found in the frequency of eating out among adult males and females [31]. The results of other studies and those of our study do not exactly match because of the difference in demographic characteristics of our study sample. University students are not similar to working individuals, who usually eat their lunch away from home most of the time because their place of work is far [28]. Additionally, females, compared to males, are more likely to go home during weekends. Our study showed that those who went home during weekends, compared to those who stayed in dormitories, were more likely to eat out.

Taste, convenience, and the opportunity to meet with friends have been found to be common reasons for eating out [32]. The same was observed for university students in our study. The opportunity to meet friends was the most common reason for eating out. In addition, most junior university students usually eat in the canteen, which offers a small selection of dishes; thus, the second most common reason for eating out was to improve their diet via adding variety to it.

Street food is defined as ready-to-eat food and beverages, processed or fresh [33]. This kind of food has received ample attention for its role in income generation and hygiene quality [34]. The types of food sold in bistros (i.e., small restaurants) are similar to street food, but one can buy more of this type of food in a single bistro. Bistros are the best place for a university student to improve their diet and save money. The hot pot constitutes a source of popular Asian dining culture. It dates back to a thousand years ago in Mainland China. It has become a favorite in many cities, especially Chongqing. Hot-pot restaurants are also becoming increasingly popular among university students in Chongqing, China. They are also popular among Chinese adults aged 18–59; modern restaurants, such as Western style fast-food restaurants and bakeries, are not the most common places for eating out [28].

As shown in our study, a small number of individuals pay attention to nutrition and food safety when eating out. They pay particular attention to the characteristics of dishes and prices. The same condition applies to ready-to-eat snacks in China, where price is still a primary factor that prevents people from eating out frequently [35]. Reduced nutrition and food safety concerns are considered significant factors associated with the frequency of eating out [12].

Many individuals are aware that eating out for a long time is bad for health. They are also aware of the potential risks involved in eating too much unhygienic, fried, barbecued, and spicy food. The possible reasons for these risks are too much unhygienic food, lack of nutrition and food safety knowledge on eating out, picky eating, and economic factors. Picky eating has also been cited as a barrier to family meals across income statuses and is a factor that needs to be addressed clearly to change patterns [36,37]. Thus, the majority of the present study’s participants expressed a high demand for nutrition and food safety knowledge when eating out. However, only a minority reported using this information when restaurants are selected. Regarding eating as a social event in the presence of family and friends can inevitably also affect food choices [38,39,40]. Because of a lack of nutrition and food safety knowledge, nutrition and security will not be the first consideration when eating out. Additionally, long-term habits of eating out and peer dietary preferences might cause one to ignore nutrition and food safety when choosing a restaurant. Educational interventions to improve knowledge related to eating out are needed in order to promote healthy eating out behaviors among Chinese university students [41].

Environmental factors impact the frequency of eating out among Chinese university students in the same way that they impact snacking among children. The trend of eating out frequency and the trend of snacking are both sensitive to income dynamics. The higher the monthly living expenses are, the higher the frequency of eating out is. The trend of monthly accommodation fee is similar to that of living expenses. Families with a low-income report that eating at home is a means to save money [38]. For university students, eating in canteens is a means to save money. One study found that no significant difference exists between low and high consumers of out-of-home prepared food in terms of the prevalence of overweightness, obesity, and truncal adiposity [42]. In our study, the BMI of participants was not found to be a factor that affected the frequency of eating out. A study in China has shown that eating out is not associated with being overweight and obesity in women [27].

This study has certain limitations. This study only included students from four universities in the Chongqing University Town, so the current findings may not be generalizable to the whole population of adults in China. Further studies with a larger sample size that covers adults of other ages are needed. Moreover, information regarding eating out was collected by self-report, which might have introduced response bias to the current findings. Owing to the cross-sectional survey data used in the current study, no causal inferences regarding the results can be made.

## 5. Conclusions

Our study revealed that the frequency of eating out is relatively high among Chinese university students. The frequency of eating out among female students is higher than that among male students during weekdays. The higher the monthly living expenses are, the higher the frequency of eating out is. Many potential risks are involved in eating out, and the majority of participants expressed a high demand for nutrition and food safety knowledge when eating out. Based on the study results, government may take intervention strategies to reduce or change eating out behavior by considering this issue from a student’s perspective.

## Figures and Tables

**Table 1:** Demographic characteristics of the study population by gender (*n* = 4236).

Demographic Variables	Total (*n* = 4236)	Male (*n* = 1749)	Female (*n* = 2487)
	*n* (%) or Mean (SD)	*n* (%) or Mean (SD)	*n* (%) or Mean (SD)
**Age**	19.8 (1.2)	19.9 (1.3)	19.7 (1.2)
**BMI**			
Thin (<18.5)	883 (20.8)	231 (13.2)	652 (26.2)
Normal (18.5–23.9)	3054 (72.1)	1304 (74.6)	1750 (70.4)
Overweight or obese (≥24)	299 (7.1)	214 (12.2)	85 (3.4)
**Residence**			
Urban	1909 (45.1)	765 (43.7)	1144 (46.0)
Rural	2327 (54.9)	984 (56.3)	1343 (54.0)
**Father’s education level**			
Primary school or below	669 (15.8)	289 (16.5)	380 (15.3)
Middle school	1718 (40.6)	710 (40.6)	1008 (40.5)
High school/secondary	1204 (28.4)	501 (28.6)	703 (28.3)
College education or more	645 (15.2)	249 (14.2)	396 (15.9)
**Mother’s education level**			
Primary school or below	1152 (27.2)	492 (28.1)	660 (26.5)
Middle school	1668 (39.4)	704 (40.3)	964 (38.8)
High school/secondary	1013 (23.9)	407 (23.3)	606 (24.4)
College education or more	403 (9.5)	146 (8.3)	257 (10.3)
**Monthly living expenses (CNY)**			
<800	1159 (27.4)	521 (29.8)	638 (25.7)
800–1200	2164 (51.1)	862 (49.3)	1302 (52.4)
>1200	913 (21.6)	366 (20.9)	547 (22.0)
**Monthly accommodation fee (CNY)**			
<600	2698 (63.7)	949 (54.3)	1749 (70.3)
600–900	1295 (30.6)	661 (37.8)	634 (25.5)
>900	243 (5.7)	139 (7.9)	104 (4.2)
**Living place while studying**			
Dormitory	3948 (93.2)	1623 (92.8)	2325 (93.5)
Home	216 (5.1)	85 (4.9)	131 (5.3)
Renting house outside	72 (1.7)	41 (2.3)	31 (1.2)
**Living place during holidays**			
Dormitory	2699 (63.7)	1187 (67.9)	1512 (60.8)
Home	1042 (24.6)	350 (20)	692 (27.8)
Renting house outside	495 (11.7)	212 (12.1)	283 (11.4)

Note: Data was expressed as mean (SD) for continuous, *n* (%) for other variable; SD: standard deviation.

**Table 2:** Frequency of eating out per week for the last month by sex (*n* = 4236).

Frequency of Eating out	Total (*n* = 4236)	Male (*n* = 1749)	Female (*n* = 2487)	*p*
	*n* (%)	*n* (%)	*n* (%)	
Weekdays				0.629
<3 times	3115 (73.7)	1279 (73.3)	1836 (74.1)	
≥3 times	1110 (26.3)	465 (26.7)	645 (25.9)	
Weekend				0.04 *
<3 times	3043 (72.6)	1284 (59.2)	1759 (60.8)	
≥3 times	1150 (27.4)	445 (25.8)	705 (28.6)	

Note: * *p* < 0.05 (significant difference).

**Table 3:** Logistic regression for the factor effects of the frequency of eating out.

**Variable**	**Frequent Eating Out during Weekdays**			
	***p*-Value**	**OR ^†^**	**95% CI ^†^**	
Monthly living expenses (CNY)	<0.001 **			
<800	<0.001 **	0.366	0.281–0.475	
800–1200	<0.001 **	0.673	0.555–0.475	
>1200		1		
Monthly board expenses (CNY)	<0.001 **			
<600	<0.001 **	0.494	0.361–0.475	
600–900	<0.001 **	0.572	0.425–0.475	
>900		1		
Living place during holidays	<0.001 **			
Renting house outside	<0.001 **	1.654	1.303–0.475	
Dormitory	0.149	0.878	0.736–0.475	
Home		1		
**Variable**	**Frequent of Eating Out during the Weekend**			
	***p*-Value**	**OR ^†^**	**95% CI ^†^**	
Monthly living expenses				
<800	<0.001 **	0.324	0.250–0.420	
800–1200	<0.001 **	0.563	0.464–0.683	
>1200		1		
Monthly accommodation fee	0.023 *			
<600	0.006 *	0.640	0.466–0.880	
600–900	0.018 *	0.693	0.512–0.939	
>900		1		
Living place during holidays	<0.001 **			
Renting house outside	<0.001 **	2.362	1.869–2.985	
Dormitory	0.029 *	0.823	0.690–0.980	
Home		1		

Notes: * Statistically significant; ** statistically significant (*p* < 0.01); **^†^** OR: odds ratio; CI: confidence interval.
